# Car sunshade-induced craniofacial injury: a case report

**DOI:** 10.1186/1752-1947-5-175

**Published:** 2011-05-10

**Authors:** Mahdi Sharif-Alhoseini, Hadi Khatibi, Mojtaba Chardoli, Vafa Rahimi-Movaghar

**Affiliations:** 1 Sina Trauma and Surgery Research Center, Sina Hospital, Tehran University of Medical Sciences, Tehran, Iran; 2 Donya-e-Khodro Weekly, Tehran, Iran; 3 Emergency Department of Hazrat-e-Rasool Hospital, Tehran University of Medical Sciences, Tehran, Iran; 4 Research Centre for Neural Repair, University of Tehran, Tehran, Iran

## Abstract

**Introduction:**

We report the case of a man who sustained a craniofacial injury after spontaneous lateral airbag deployment resulting in his face being struck by a car sunshade. This highlights the potential damage that can be caused by any object placed between a lateral airbag and a car occupant.

**Case presentation:**

We report the case of a 33-year-old Caucasian man who was the driver in a frontal collision. He had opened the car sunshade and turned it 90° towards the left. As he was driving, he struck a bus, causing the driver's lateral airbag to spontaneously deploy. The airbag pushed the sunshade against his face and injured him.

**Conclusions:**

Car sunshades can cause significant craniofacial injury. We suggest that sunshade design must be improved to reduce the risk of potential injuries to car occupants. We recommend a new, safer sunshade design.

## Introduction

Although there are reports of injuries caused by airbags [1-5], we are unaware of any literature describing injuries from car sunshades. We report a case of severe craniofacial injury after spontaneous lateral airbag deployment that caused the sunshade to strike the driver's head. We also discuss the mechanism of sunshade induced injuries.

## Case presentation

**Figure 1:**
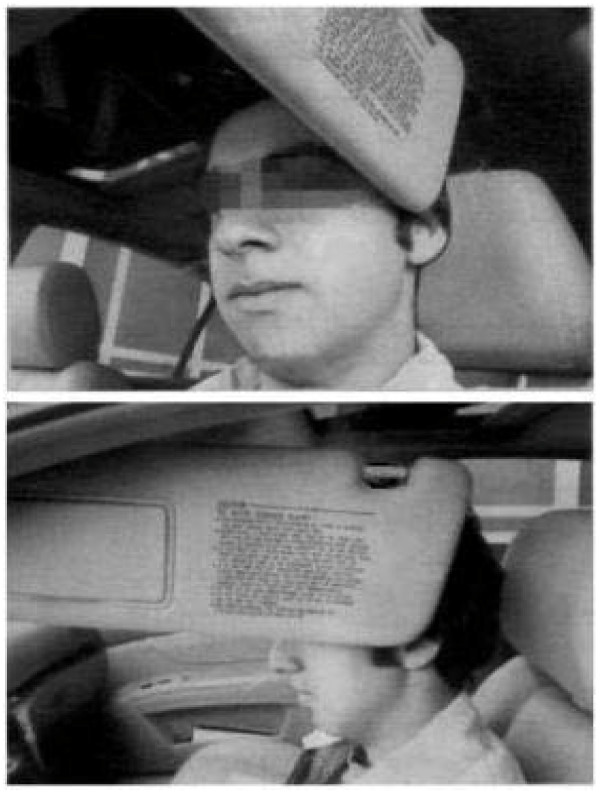
**Lateral airbag deployment pushed the sunshade against driver's face**. The sunshade was completely deformed and caused injury to the left side of the temporal skull, orbit, face, and nose. This figure is a model, and the model has provided informed written consent to his image being used.

A 33-year-old Caucasian man was referred to Shahid-Rasi Hospital in Shahindezh (West Azarbaijan province, Northwestern Iran) after a motor vehicle crash. He was driving on a two-way mountain road in a south-north direction before sunset. Because of sunlight, he opened the car sunshade and turned it 90° towards the left. As he was driving at 60 km/hour on a sharp curve in the road, his car suddenly hit a bus coming from the opposite direction. The driver's lateral and steering wheel airbags spontaneously deployed. The lateral airbag pushed the sunshade against his face so hard that the sunshade was completely deformed and caused injury to the left side of the face (Figure 1). He suffered abrasions on the left side of the face, retinal damage, and fractures of the skull base and nose. He also suffered superficial right forearm burns due to the rupture of the steering wheel airbag. His left hand was caught in the steering wheel, resulting in left distal radial and ulnar fractures. He underwent operative fixation of his nose and wrist factures and was referred to an ophthalmologist for evaluation of his retinal injury.

## Discussion

Airbag-associated injury occurs in 43% of airbag deployments [6]. Typically, airbag-related injuries are minor, but severe or fatal injuries are also reported [7]. Minor injuries such as abrasions, contusions and lacerations are usually detected on the face, neck, chest, and upper extremities [8,9]. Airbag deployment also releases high-temperature gases, including nitrogen and carbon dioxide, and produces sodium hydroxide, a very irritating alkaline material, which can cause superficial and even full thickness burns [10,11]. As demonstrated by this case, an opened and turned sunshade can also be a potentially dangerous object between a lateral airbag and a driver or passenger.

## Conclusions

When the lateral airbag deploys, it pushes the sunshade onto the occupant's face and head. Consequently, it seems that vehicles with side airbags should not have moveable sunshades that can be placed in the lateral position. We suggest the design and use of sunshades that do not project into the vehicle or the use of sunglasses.

## Consent

Written informed consent was obtained from the patient for publication of this case report and any accompanying images. A copy of the written consent is available for review by the Editor-in-Chief of this journal.

## Competing interests

The authors declare that they have no competing interests.

## Authors' contributions

MS wrote the case report and performed the literature search. HK wrote the Farsi version of the draft and organized the photographs. MC and VRM designed the methodology, discussed, and edited the draft. All authors read and approved the final manuscript.
